# An integrated multi-hazard assessment using machine learning in the complex terrains of Northern Pakistan

**DOI:** 10.1038/s41598-026-41029-w

**Published:** 2026-03-01

**Authors:** Muhammad Ismail Khan, Muhammad Shafique, Ghazanfar Ali Khattak, Abid Yahya, Irfan Anjum Badruddin

**Affiliations:** 1 National Centre of Excellence in Geology, University of Peshawar, Peshawar, Pakistan; 2 GIS and Space Application in Geosciences (GSAG) Lab, National Center of GIS and Space Application (NCGSA), Islamabad, 44000 Pakistan; 3 Department of Electrical and Communications Systems Engineering, Botswana International University of Science and Technology, Palapye, Botswana; 4 Department of Mechanical Engineering, College of Engineering, King Khalid University, Abha, 61421 Saudi Arabia

**Keywords:** Multi-hazard assessment, Machine learning, AHP, Northern Pakistan, Risk assessment, Climate sciences, Environmental sciences, Environmental social sciences, Hydrology, Natural hazards

## Abstract

The increasing frequency of natural hazards, intensified by climate change, poses substantial challenges to sustainable development worldwide. Northern Pakistan, particularly the Hunza district, is highly susceptible to multiple hazards, including landslides, earthquakes, glacier-induced floods, debris flows, and Glacier Lake Outburst Floods (GLOFs), driven by both climatic and tectonic factors. A multi-hazard assessment is essential to understand the complex interactions between these hazards, offering a comprehensive perspective on risk and facilitating more effective disaster preparedness and mitigation strategies. This study addresses the existing gap in multi-hazard assessments, which are often confined to single-hazard evaluations, by developing an integrated multi-hazard susceptibility map for the Hunza district in Northern Pakistan. The region’s complex topography, active tectonics, and accelerated glacier melting contribute to its high vulnerability to cascading and co-occurring hazards. The integrated assessment utilizes diverse data sources, including topographic attributes, geological, hydro-meteorological, environmental variables, and literature-derived hazard map for multi-hazard susceptibility analysis. A Machine Learning (ML) Forest-Based Classification and Regression (FBCR) model, Analytical Hierarchy Process (AHP), and Vs30-based site characterization was employed to classify and generate hazards individually and as integrated multi-hazard susceptibility map. The model incorporates eighteen geo-environmental variables for individual hazards assessment. The resulting multi-hazard susceptibility map indicates that 23.11% of the area is prone to landslides, 6.07% to flash floods, 4.66% to debris flows and flash floods, and 3.98% to a combination of flash floods, landslides, and debris flows. The highest multi-hazard zone, comprising seismic hazard, debris flows, landslides, and flash floods, covers 2.88% of the area, whereas low-hazard zones constitute 56.84% of the region. The landslide susceptibility model classifies 20% of the area as very high susceptible, while the flash flood, debris flow, and seismic hazard models indicate 5, 2, and 13% of the area, respectively, fall under very high susceptibility/hazard. This integrated multi-hazard approach provides a comprehensive risk assessment framework, supporting evidence-based disaster risk reduction policies and infrastructure planning in hazard-prone regions. The findings identify critical high-hazard zones, offering data-driven insights for targeted mitigation strategies and disaster risk reduction efforts.

## Introduction

The compounded effects of multiple hazards, intensified by their increasing frequency under climate change, pose substantial challenges to sustainable development worldwide. Climate change has altered environmental patterns, leading to erratic precipitation, temperature fluctuations, and accelerated glacier melting. These changes have intensified the frequency and magnitude of natural hazards such as landslides, debris flows, floods and Glacial Lake Outburst Floods (GLOF), resulting in widespread damage to communities, infrastructure, and economies^1^. Dilley et al.^2^ reported that globally around 30 million km^2^ of land and over 4.3 billion people are exposed to at least one natural hazard, 3.5 million km^2^ area and 700 million people are exposed to two hazards and 0.5 million km^2^ area and 105 million people are exposed to three or more hazards. Despite the significant risk from multiple hazards, hazard assessment studies have predominantly focused on single hazards, thereby underestimating the overall risk posed by multi-hazard scenarios, which may co-occur or trigger cascading effects^3^. Moreover, given the population growth, urbanization and deteriorating economic conditions particularly in developing countries, the vulnerability of infrastructure and already marginalized mountain communities is on the rise^4^. Consequently, multi-hazard risk assessments are critical and gaining momentum in hazard-prone countries to utilize for developing disaster risk reduction policies and measures^5^.

Within this global context, Pakistan ranks among the most disaster-prone countries in South Asia, having incurred substantial social, physical, environmental, and financial losses over recent decades^6,7^. Given its complex tectonic, topographic and climatic conditions, the country has experienced and is still exposed to multiple hazards, including landslides, debris flows, floods, GLOFs, and earthquakes^8^. Intensifying climate variability, accelerating glacier retreat, and unplanned development within hazard-prone terrain have further amplified exposure, vulnerability and risk^7,9–11^. Between 2005 and 2025, more than 91,000 people lost their lives to disasters across Pakistan, resulting in economic damages exceeding $34 billion-of which approximately $10 billion stemmed from the 2010 floods and $5 billion from the 2005 earthquake and associated landslides^12,13^.

In particular, northern Pakistan bears the most direct and severe impact of these climatic and tectonic stresses. During the monsoon season (July-September), intense precipitation and high temperatures frequently trigger multiple hazards throughout the Hindukush-Karakoram-Himalaya (HKH) mountain ranges, causing extensive human losses, infrastructure destruction, and socio-environmental degradation^14,15^. Rising temperatures-averaging an increase of about 0.85 °C since 1980-have shifted monsoon patterns northward and westward in Pakistan and accelerated glacier melt, amplifying the frequency and magnitude of extreme events. Topographically, deeply incised valleys, steep slopes, and extensive glaciofluvial and alluvial landforms enhance susceptibility to slope failures, while active fault systems generate frequent small- to large-magnitude earthquakes that often trigger cascading landslides and debris flows. A prominent example is the 2010 co-seismic Attabad rockslide, which dammed the Hunza River to form a 27 km-long lake, resulting in 19 deaths, the displacement of over 6,000 people, and the loss of vital infrastructure and agricultural land. Moreover, the region is highly exposed to flash and debris floods caused by accelerated melting of glaciers and GLOF^16,17^. Recent GLOF occurrences in June 2019, April 2020, May 2022 and August 2025, triggered by the Shisper Glacier in Hunza district, caused extensive damage to downstream communities, infrastructure, and the Karakoram Highway. These hydro-meteorological and geohazards pose ongoing challenges to multi-billion-dollar infrastructure projects under the China-Pakistan Economic Corridor (CPEC), leading to increased costs, delays, and disruptions to the sustainable supply chain along the Karakoram Highway—the only land route between China and Pakistan^18^.

Given the intense concentration of hazards in Northern Pakistan and their cascading impacts on critical infrastructure, there is a growing need for comprehensive multi-hazard assessment frameworks capable of capturing these interconnected processes. However, the varying nature, impacts, causative and triggering factors, and spatio-temporal dynamics of different disasters make their integration into a single multi-hazard assessment highly complex and challenging^19^. Several methodologies have been developed for integrated multi-hazard mapping using multivariate statistical and decision-support approaches, such as logistic regression and the Analytical Hierarchy Process (AHP)^20–22^. More recently, machine-learning (ML) techniques, including tree-based algorithms such as Random Forest, have been increasingly adopted to capture complex and nonlinear relationships between conditioning and triggering factors in multi-hazard assessments^23^. These ML approaches have advanced susceptibility mapping by improving classification accuracy, handling large datasets efficiently, and facilitating more realistic and automated hazard assessments^24^.

However, Northern Pakistan has witnessed and been exposed to multiple hazards; therefore, an integrated multi-hazard assessment is urgently required to capture the region’s compounded risk environment^15^. Therefore, this study aims to fill this crucial gap and develop an integrated multi-hazard map of the major hazards (flash flood, landslide, earthquake, debris flow, and GLOF) in the Hunza district of northern Pakistan, using a machine learning algorithm to contribute to evidence-based policymaking for effective disaster risk reduction.

## Materials and methods

### Description of study area

**Fig. 1:**
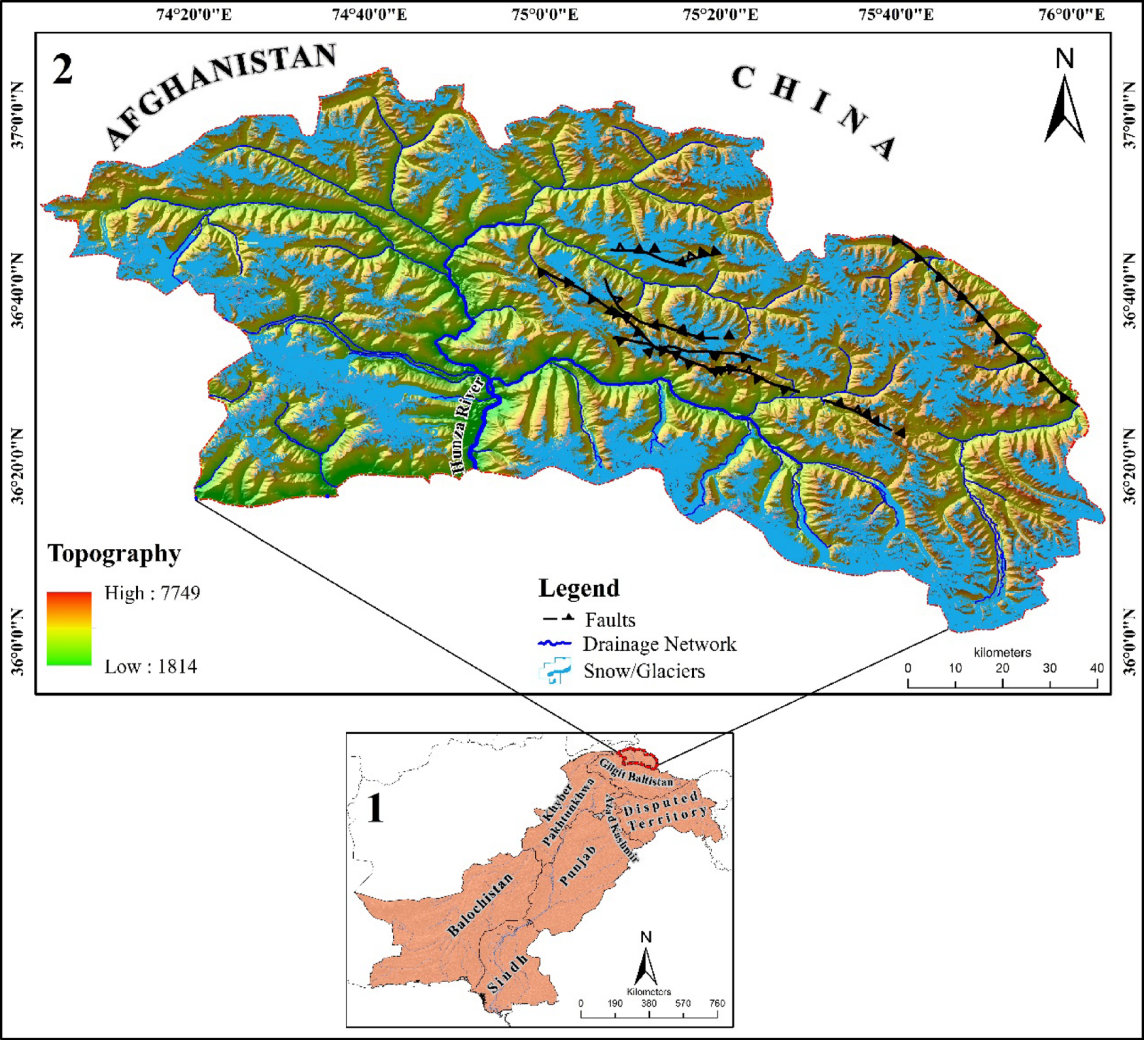
Administrative boundaries were obtained from open-source repositories Diva GIS (https://diva-gis.org) and manually adjusted for accuracy, (2) Location map of the district Hunza, generated in this study using ArcGIS Pro 2.8.3 software (https://www.esri.com/en-us/home).

The study area encompasses the Hunza district in northern Pakistan, a region characterized by its distinct topography, active tectonic settings, diverse geological features, and climatic conditions that contribute to the occurrence of multiple hazards, including earthquakes, landslides, debris flows, floods, and GLOFs. Geographically, the Hunza district is divided into three main regions: Upper Hunza (Gojal), Central Hunza, and Lower Hunza (Shinaki) (Valley and Dish, 2016). With a total area of 11,440 km^2^, elevation range of 1814 to 7749 m above sea level, and hosting some of the largest valley glaciers, including Batura (59 km), Passu (24 km), Gulkin (18 km), Hispar (61 km), and Ultar (10 km) (Fig. 1, Panel 2). The Hunza River, which drains the region, is primarily fed by the melting of surrounding glaciers and serves as a major tributary of the Indus River. As a critical water resource, it plays a vital role in sustaining downstream water availability, making it essential for irrigation, hydropower, and overall water security in Pakistan^25^. However, the onset of summer accelerates the melting of glaciers and increases the water flow in streams and rivers, accelerating erosion, debris flows and landslides^26,27^.

The study area is located in the region with the most active tectonics, a significant portion of Hunza Valley lies on the uplifted portion of the Main Karakorum Thrust (MKT) faults^28^, caused by the collision of the Indian and Eurasian tectonic plates^29^. Frequent earthquakes and active tectonic activity have extensively fractured the geological formations, making them prone to landslides. The geomorphology of the Hunza district consists of glacial-fluvial terraces, old glacier moraines, loose material on steep scree slopes, debris flow in the debris cone and colluvium deposits with split terrains, creating talus deposits at the bottom of towering cliffs^30^.

The distinctive geological setting, characterized by thrust faults, fractured rock formations, and exposed geomorphology^30^, combined with human interference, including deforestation and infrastructure development^31^, have led to frequent and devastating disasters. To inform evidence-based policies for disaster risk reduction, a comprehensive multi-hazard analysis is essential for continuous monitoring and the implementation of proactive mitigation measures against natural hazards.

### Materials

To acquire the topographic information, the ALOS-PALSAR digital elevation model (DEM) was utilized to compute the selected topographic attributes including terrain slope, aspect, plan curvature, profile curvature, Topographic Wetness Index (TWI), Relief Ratio (RR), Melton Ratio (MR), Stream Power Index (SPI), Sediment Transport Index (STI) and to extract streams and watershed boundary (Table 1). The distance from the river/stream and road was derived and classified into six buffer zones within ArcGIS Pro Environment^32^. Mean monthly rainfall data were acquired from the Global Precipitation Measurement (GPM) (https://gpm.nasa.gov/data/directory) at a spatial resolution of 0.1° (10 km) from 2014 to 2021. The rainfall data was resampled to 12.5 m in ArcGIS Pro-Platform to maintain spatial consistency with other predictor variables; however, this resampling does not increase the intrinsic precision of the rainfall data. Instead, it performs spatial averaging or interpolation to align datasets on a common grid, which is essential when integrating multiple variables. Rainfall data from 2014 to 2021, available from GPM provides adequate temporal coverage for analyzing variability and trends, given the lack of reliable meteorological stations in Hunza. A landcover map and Normalized Difference Snow Index (NDSI) were prepared based on the Sentinel-2 satellite images with 10 m spatial resolution, using the Support Vector Machine algorithm with a Kappa value of 0.85. The same image is used to compute the Normalized Difference Vegetation Index (NDVI) map. The lithology map was digitized from the geological map of northern Pakistan developed by Searle et al.^33^. GLOF hazard map was acquired from Ullah et al.^34^, with copyright clearance obtained through the publisher’s formal permissions process.

**Table 1:** Details of geo-environmental factors used in multi-hazard susceptibility mapping, including their sources, scale, and the data from which they are derived.

Data type	Factors	Data used	Sources and scale
Topographical	1. Elevation 2. Slope 3. Aspect 4. Plan Curvature 5. Profile Curvature 6. TWI 7. Streams 8. RR 9. MR 10. SPI 11. STI	PALSAR DEM	http://www.eorc.jaxa.jp/ALOS/en/aw3d30 (12.5 m*12.5 m resolution)
Geological hydro-meteorological	12. Lithology 13. Faults 14. Rainfall 15. Roads	Geological Map, GPM Data, Google Earth Pro	Geological Survey of Pakistan https://gpm.nasa.gov/data/directory (0.1°) (2014–2021 Period)
Environmental	16.Landcover 17. NDSI 18. NDVI	Sentinel 2 A-2B (ESA)	ESA Copernicus Open Access hub https://scihub.copernicus.eu/
GLOF hazard map			^34^

### Methods

#### Multi-hazard modeling using machine learning

**Fig. 3:**
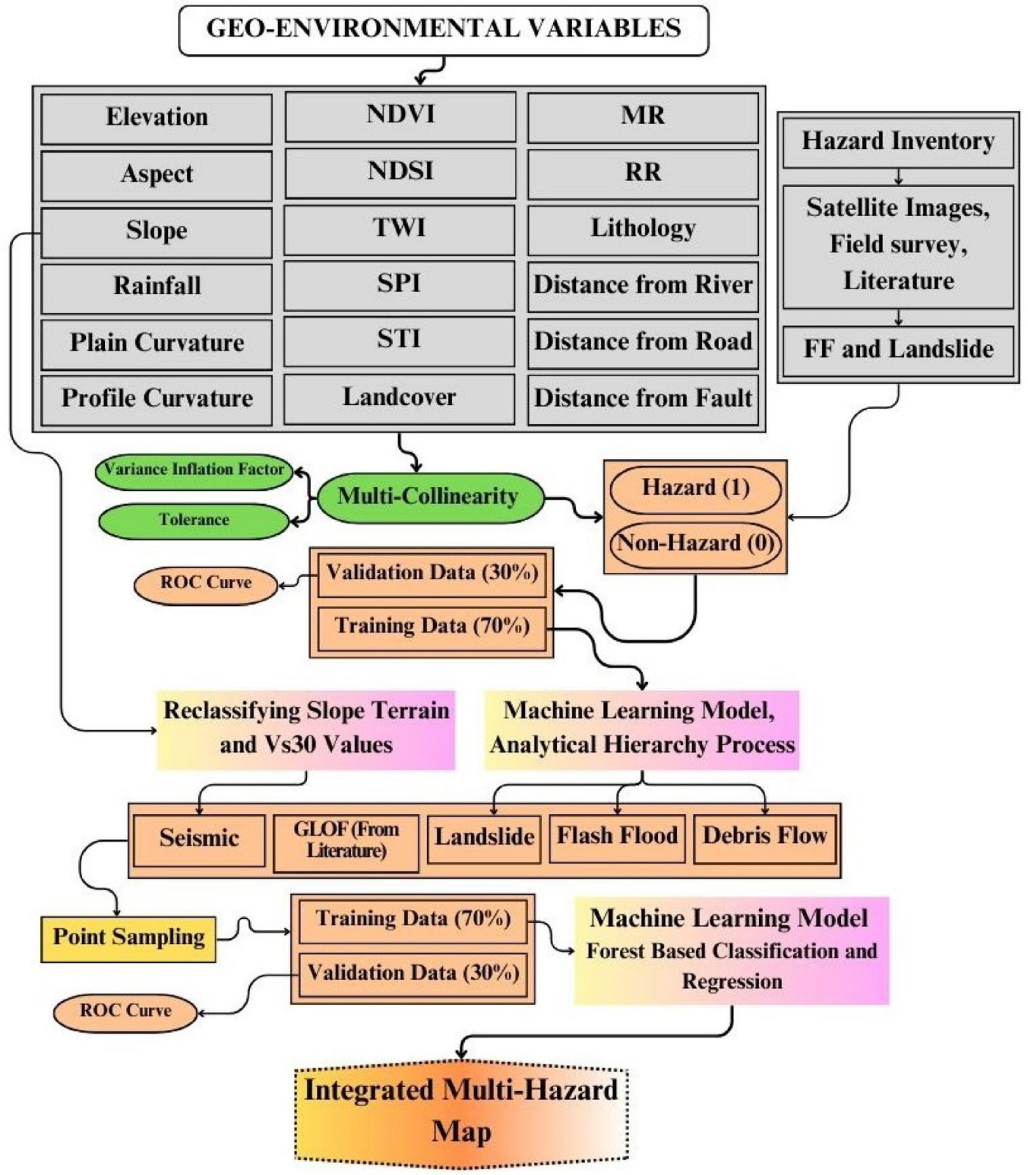
Methodology for developing the multi-hazard susceptibility maps.

In this study, we have integrated five hazards, including Landslide (LS), Debris Flow (DF), Flash Flood susceptibility (FFS), Seismic hazard (SH) and GLOF, using Forest-Based Classification and Regression Model (FBCR) (Fig. 3), an adaptation of Breiman’s random forest algorithm^35^. FBCR is a supervised machine-learning method that combines multiple decision trees, with each decision tree casting a unit vote for the most popular class^36^. Each tree in the forest is built using a bootstrap sample from the data, which may include duplicates and omit some original observations^37^. The FBCR is a natural, non-linear modeling tool that estimates variable importance, generalization error rates through the out-of-bag (OOB) error, and contributions to total risk^38^. The algorithm’s power lies in random feature selection and bagging^36^. As a result, FBCR can tackle both classification and regression problems, making it a popular choice for natural hazard forecasting^39^. The algorithm of FBCR is based on tree-structured classifiers. Each tree $\:{h}_{k\:\left(x\right)}$ is trained on a bootstrap sample of the data, and the final prediction $\:{H}_{\left(x\right)}$.

is obtained by aggregating the outputs of all trees as follows:

1 $$H_{{\left( x \right)}} = \frac{1}{n}\mathop \sum \limits_{{k = 1}}^{n} h_{k} \left( x \right)$$

where $\:x$ is the vector of hazard-related predictor variables, $\:{h}_{k}\:$is the prediction of the $\:\left(x\right)\:$tree, and *n* is the total number of trees in the FBCR model.

**Fig. 4:**
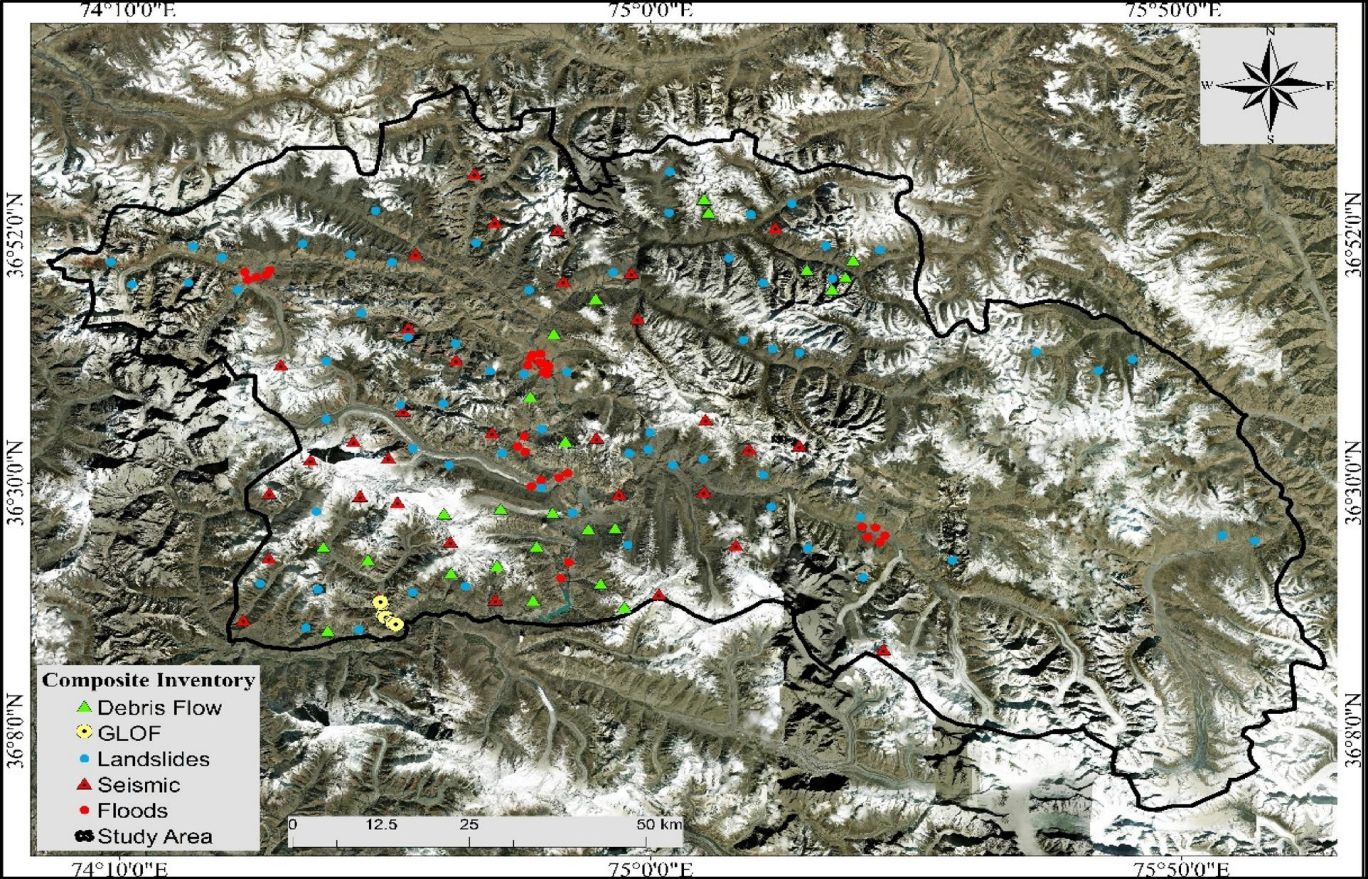
Composite multi-hazard inventory map developed for this study by the first author using ArcGIS Pro 2.8.0 software, showing sampled locations from each higher-hazard zones of individual hazard map.

Moreover, to integrate multiple hazards, each hazard map was classified (1 representing very low hazard, 2 for low hazard, 3 for moderate hazard, 4 for high hazard, and 5 for very high hazard (Fig. 16) and standardized to a common classification framework^40^. This ensured comparability between hazard maps derived from different modelling approaches (ML and AHP) and prevented any single hazard from dominating the integrated map due to differences in their native scales. Prior to integration, hazard weights derived from mean decrease Gini (MDG) analysis (details given in Sect. index importance degree) performed after model training, and further informed by field surveys, literature review and expert input, were applied to calibrate the combined map. All maps were classified and standardized to harmonize hazard-specific classification schemes, thereby ensuring consistency for direct comparison and subsequent analytical integration. The susceptibility modeling technique applied in this study requires samples of both hazards and non-hazards to generate multi-hazard susceptibility maps^41^. Therefore, an equal number of hazard and non-hazard locations was digitized in point format from each hazard map, and was assigned a value: (1) as hazard and (0) as non-hazard, which simplified the classification and integration process^15,24^. Following Pourghasemi, et al.^14^, Yousefi, et al.^15^, Pourghasemi, et al.^41^, the hazard locations were determined by identifying areas on each hazard map that showed the highest susceptibility levels, such as high and very high classes, while non-hazard locations were randomly sampled from moderate and low susceptibility classes. Random sampling also ensures the dataset’s spatial distribution throughout the study area, providing a balanced input for further processing. A total of 120 points were defined as LS points, while 120 as non-LS points, 90 points as SH, and 90 as non-SH points, 33 GLOF hazards points and 33 non-GLOF, 50 points for DF-50 non-DF, 60 FF points, while 60 represents non-FF points, and finally, the composite inventory of hazard locations was created (Fig. 4).

The number of hazard points varies due to the spatial distribution and intensities of each hazard map class. GLOF hazard is limited to a small region within particular glaciers, resulting in fewer points. LS is a frequent hazard across the district, leading to more hazard points. The valley and alluvial fans in the region are highly exposed and frequently impacted by debris flows, particularly during the rainy season or due to the accelerated triggering of glacial activity. Additionally, we divided the hazard and non-hazard point data using a random partitioning into two chunks of train and test by the ratio of 70 and 30%, where the train chunk was used for training, model tuning, and determining the variable’s importance, while the test chunk was used for validation^42,43^. Several rates, like 80/20 and 60/40 were tried to determine which provides the best model accuracy. However, 70/30 showed higher precision; thus, we selected 70/30 to construct the ML model. The point dataset was further utilized to extract the raster values of each hazard map as the input features (Predictor) for training the machine learning-FBCR model. These predictors enable the model to identify the spatial patterns between hazards and reflect the probability of hazard occurrence. Moreover, the predictor variables were exported from ArcGIS Pro 2.8.3 and processed in Python (scikit-learn), where the “RandomforestClassifier” was used to build the integrated multi-hazard map^44^. In addition, to reduce the bias caused by sampling in the data-sampling process, repeated sampling was performed over 100 runs, which is the standard value of the FBCR-model^45^, including the learning setting: number of trees = 500, leaf size = 5, and tree depth range = 47–50, and the number of predictors at each split was determined by the scikit-learn default (√number of predictors). However, the final map was imported and processed in ArcGIS Pro 2.8.3 software, including reclassification into hazard classes using the Natural Breaks (Jenks) method.

#### Using out-of-bag estimates to monitor error

We used the out-of-bag (OOB) method to estimate the generalization error of the model. By adjusting the number of decision trees, we observed a decrease in OOB error, which stabilized with minimal fluctuation beyond a sufficient tree count^46^. Lower OOB error indicates better model accuracy. Additionally, the OOB error was calculated as the ratio of misclassifications to the total samples^47^. Moreover, the OOB is constructed following the Eq. (2).

2 $$\:MSE={n}^{-1}{\sum\:}_{i=1}^{n}\left({t}_{i-\genfrac{}{}{0pt}{}{-}{t}}\right)$$

where $\:{t}_{i}$ is the average of all OOB predictions, n is the number of OOB observations in each tree, and MSE is the mean square error obtained during the construction of the classification trees^45^.

### Individual hazard assessment and influencing factors

#### Flash flood and landslide inventory

Hazard inventory is crucial in ML models and reveals the spatial interaction between the location of historical hazards and geo-environmental factors^48^. Therefore, in this study, an extensive flash flood and landslide inventory was developed (Fig. 5), comprising 56 flood-prone locations developed using Google Earth Pro, literature and field observations following Abbas, et al.^49^. Similarly, the landslide inventory consists of 344 locations, manually digitized through the visual interpretation of multi-temporal high-resolution Google Earth images (2010–2023), and verified during the field survey in September 2024^50^. Since we used the binary classification-FBCR model to develop the FFS and LS map, the model also requires the non-hazard locations (detail methodology is given in Sect. 2.3.1)^51^, which were sourced from areas that never experienced flooding or landslides, such as flat or gently sloping regions.

**Fig. 5:**
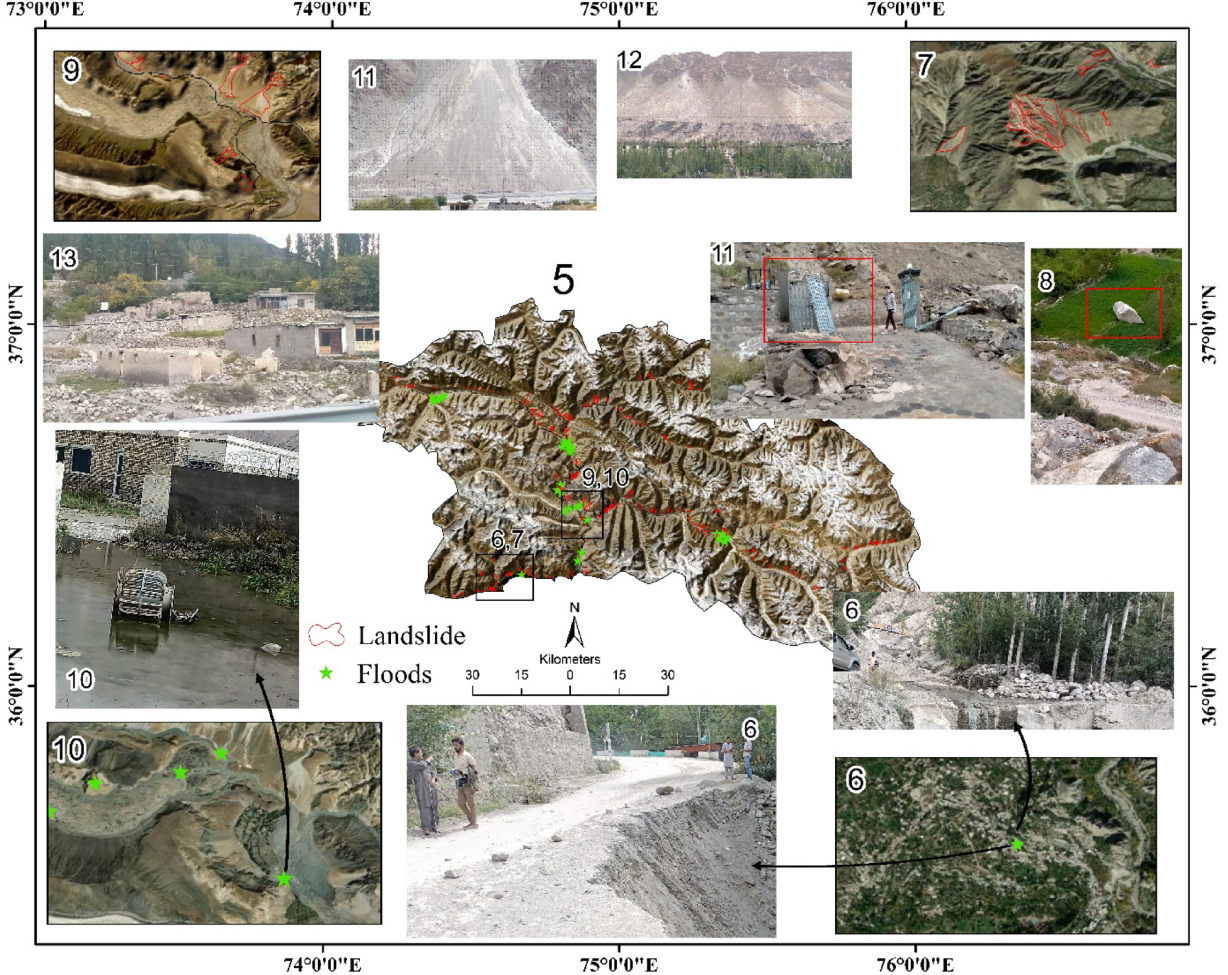
Landslide-flood Inventory map for this study generated in ArcGIS Pro 2.8.3, the first author is depicted in (Panel 6), verifying landslide location with handheld GPS during the field survey, (Panels 6, 7, 9 and 10) magnified views of the landslide-flood areas, (Panel 8) Field photograph showing displaced boulders and damaged agricultural terraces opposite the recorded landslide area in Panel 7, (Panel 11) field photograph from Hassanabad along the Karakoram Highway, near the Excise and Taxation Department Hunza, showing a damaged structure as evidence of slope failure, (Panels 11 and 12) field photographs showing landslide-affected slopes along the Karakoram Highway, (Panel 13) destroyed houses and scattered rockfall or debris flow deposits near Ghulkin village, (Panel 10) Passu Gojal area showing flood impacts, (Panel 6) Karimabad areas frequently affected by landslides-floods, as reported through casual discussions of author with local residents during field visits, with field images as evidence.

#### Influencing factors

Based on the literature review^52–54^ and the characteristics of the historical geohazards in the study area and multiple field observations, we selected eighteen geo-environmental variables for susceptibility mapping. Twelve variables, including altitude, slope, aspect, plan curvature, profile curvature, distance from river, distance from road, land use, NDVI, lithology, rainfall, and TWI were selected for FFS, out of which ten variables, such as slope, rainfall, elevation, aspect, profile curvature, distance from river, distance from road, land use, lithology, and fault, were chosen for the LS, and eight variables, slope, rainfall, TWI, Melton ratio, relief ratio, SPI, NDSI, and STI, were selected and mapped within ArcGIS Pro 2.8.3 platform for DF (Fig. 14).

The terrain aspect is an important factor in determining flood occurrence because it is directly associated with the convergence and direction of water flow. Additionally, altitude impacts the natural movement of water, and thus influences flood susceptibility^55^. Terrain slope determines the flow and velocity of runoff and significantly influences damage distribution and intensity, and its angle impacts DF, FF, and LS, with flooding common in flat areas and steep slopes prone to destabilization^53,56^. The magnitude and intensity of rainfall are the primary triggers for all three hazard occurrences^57,58^. Topographic characteristics of an area were understood by plan and profile curvature^26^. Plan and profile curvature influence FFS by affecting water flow and runoff patterns, and LS by indicating slope steepness and change rate through profile curvature^59^. Landcover and NDVI affect the distribution and impacts of flash floods, with vegetation density impacting the velocity and runoff. Concurrently, landcover modifies hydrological and geomorphological dynamics, influencing landslide occurrence by altering soil stability and erosion processes^60,61^. The lithology determines the permeability and infiltration and hence influences the surface runoff and destructiveness of flash floods^62^. Similarly, lithology and distance to faults collectively influence LS by affecting geological stability and soil strength^32^. TWI, which illustrates soil wetness, water depth, and saturation, informs topographical controls and hydrological processes for FFS, while also indicating the water-saturated zones affecting infiltration and runoff, and susceptibility to LS and DF^63^.

**Fig. 5:**
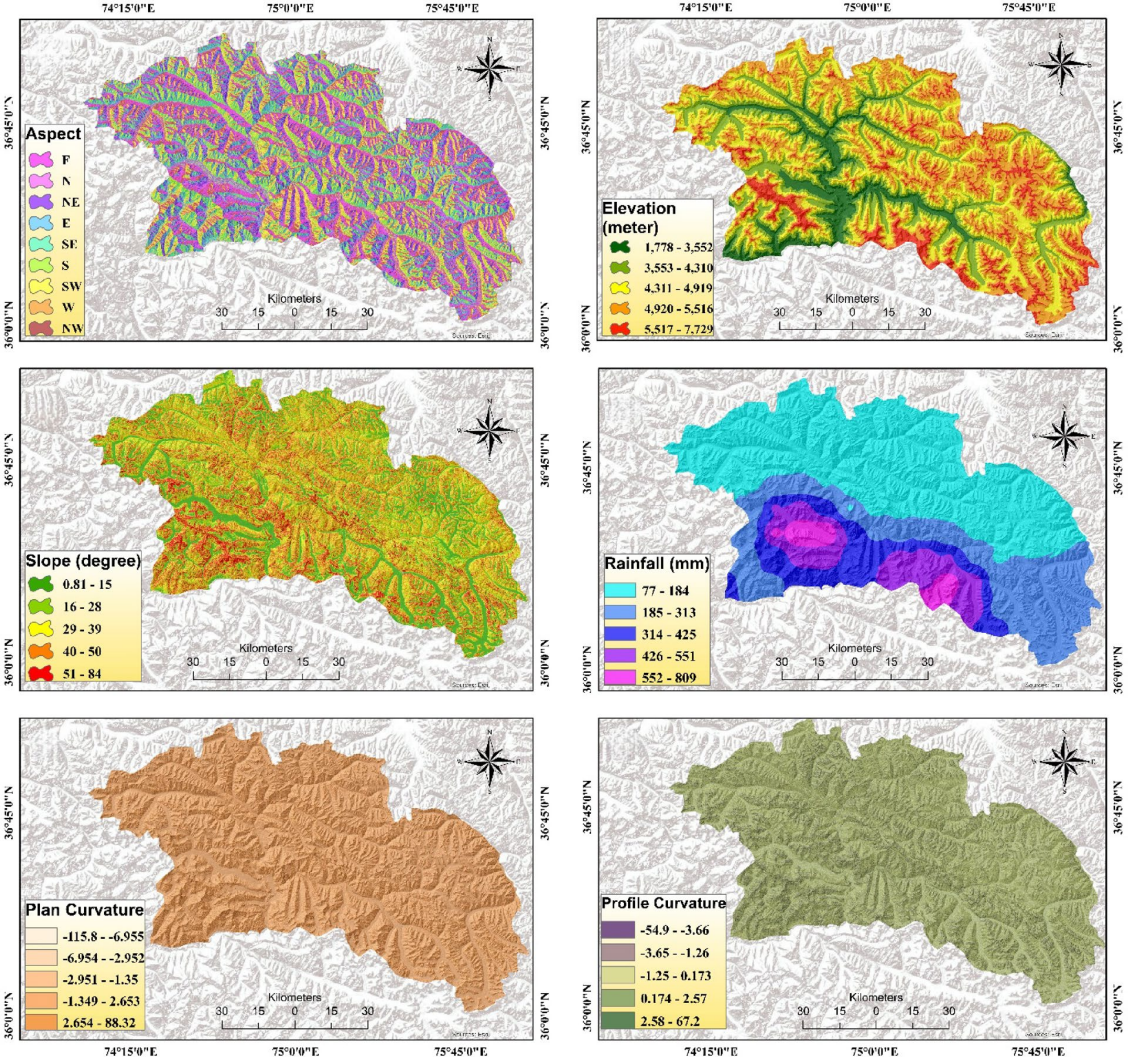

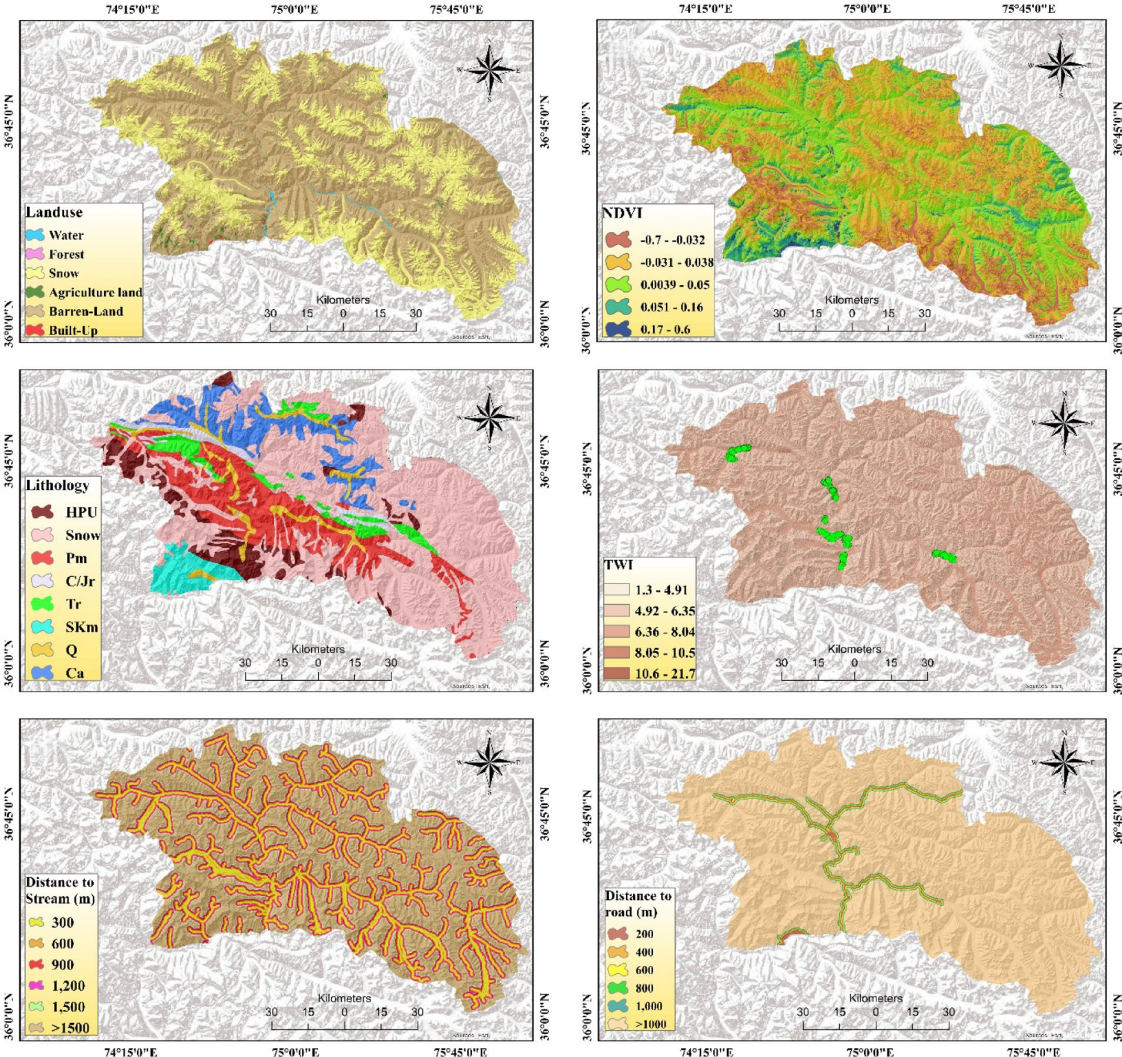

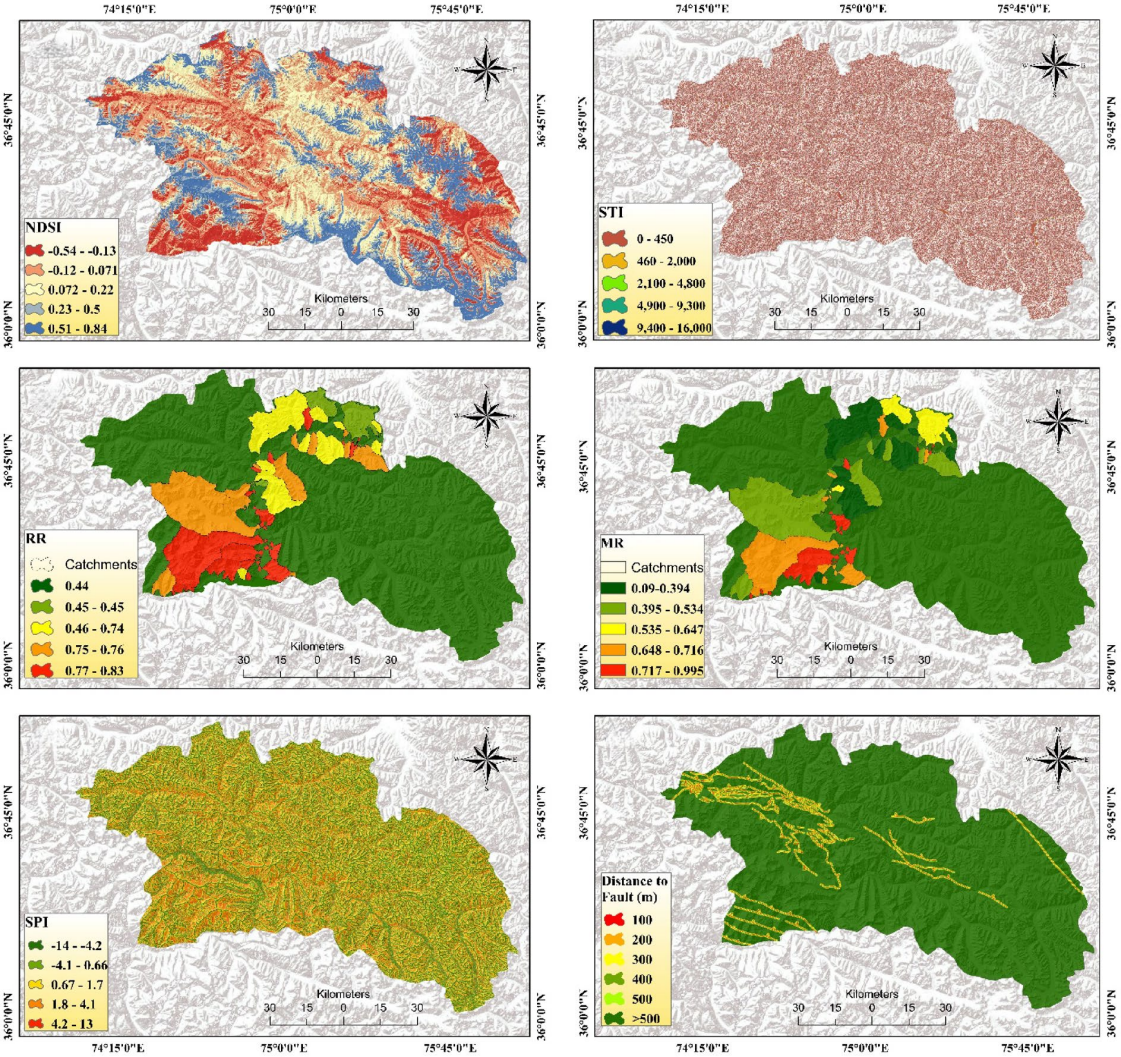
Multi-hazard susceptibility factors, computed using open-source datasets and generated in ArcGIS Pro 2.8.3 for this study.

Distance from rivers affects the probability and magnitude of flooding and LS by influencing terrestrial water storage, flood events, and destabilizing slopes through river erosion and water accumulation^57,60^. Roads built on high-altitude areas alter water flow patterns, inducing high-intensity FFS that typically destroy road infrastructure and compromise slope stability, increasing LS through changed drainage patterns and erosion^61^. The MR, a ruggedness index, indicates topographic complexity, which can facilitate DF initiation by increasing runoff concentration and erosion potential^64,65^. The RR influences DF susceptibility by increasing the likelihood of slope failure due to its direct impact on gravitational stress^65^. STI exacerbates DF potential by optimizing sediment transport and deposition, whereas the NDSI influences DF by quantifying snowmelt-driven runoff variability, thereby enhancing the likelihood of debris flow events during summer months^54,66^.

#### Debris flow hazard (DF)

The DF hazard assessment methodology follows Shah, et al.^54^ and Ullah, et al.^67^, whose study of in the same region topographically complex terrain aligns closely with the geomorphological and climatic conditions of the current study area. Given the absence of historical DF inventory data, a prerequisite for data-driven methods like ML (utilized for LS and FF with available inventories), the AHP was selected. AHP, validated in analogous data-scarce mountainous environments^68^, derives factor weights through expert-guided pairwise comparisons, with consistency ratios computed to ensure reliable judgment matrices (Fig. 5, Panel 6). The selected DF influencing factors are consistent with Shah, et al.^54^ and Ullah, et al.^67^, underwent rigorous multi-collinearity evaluation to ensure factor independence (Table 2). For a detailed description of the methodology, the reader is referred to Shah, et al.^54^ Ullah, et al.^67^.

#### Seismic hazard (SH)

Shear wave velocity of the top 30 meters surface (V_s_^30^) is one of the important indicators of the seismic response amplification and is frequently utilized for seismic site characterization around the globe^69–71^. In regions with limited field measures, the topographic slope and geological information are effectively utilized as a proxy for the V_s_^30^-based seismic site characterization^72,73^. In this study, a V_s_^30^-based seismic site characterization map is developed by reclassifying the terrain slope map using the thresholds after Allen and Wald^74^.

### Index importance degree

The FBCR model computes the relative importance of each predictor (referred to here as the ‘index importance degree’) to quantify its contribution to the modelled hazard susceptibility. In this study, variable importance was evaluated using the mean decrease in Gini impurity (MDG) of the Random Forest (RF) algorithm for the integrated multi-hazard mapping, FFS, and LS analyses. MDG measures how well a split divides samples in a node, considering feature importance and splitting frequency and is mathematically represented as:

3 $$I_{g} \left( f \right) = \sum w_{{p,n}} \sum \Delta i_{f} \left( {\tau ,M} \right)$$

where $\:{I}_{g}$ is the Gini importance, which specifies how often a specific feature is chosen to split and how important that feature is. Assigning the weight $\:{w}_{p,n}\:$defines any imbalance in the distribution of classes during the learning Algorithm. while $\sum \Delta i_{f}$ (, ) represents the change in Gini impurity resulting from splitting the data based on feature at a specific threshold τ and node *M*.

Generally, natural hazards are threshold-dependent and are influenced by various effective factors^75^. Therefore, determining the relative importance of each hazard is key to evaluating the multi-hazard risk patterns^76^. Python (scikit-learn) was used to compute the Mean MDG from the RF model^36,77^, and validated the relative importance of each hazard in the multi-hazard susceptibility map through field surveys, literature review, expert opinions, and decision-maker engagement^78^. This validation approach ensured alignment between the calculated hazard weights and real-world conditions.

### Performance metrics

#### ROC curves

Validation is an essential component in the development of susceptibility mapping and the determination of its quality^79^. The FFS and multi-hazard susceptibility maps were validated by the receiver operating characteristic (ROC) curve. The Area Under Curve (AUC) value for this was estimated considering the true positive (TP) and false positive (FP) values of susceptibility modeling. TPs were pixels that were correctly estimated to be susceptible to hazards, while FPs were pixels that were incorrectly estimated to be susceptible to hazards^61^. Further, the ROC curve with AUC values has a range of 0.5–1.0. A higher AUC indicates a better model performance^80,81^.

## Results

### Individual hazard assessment

#### Multi-collinearity analysis

To assess the multi-collinearity between the causative factors selected for the multi-hazard susceptibility assessment, we conducted a multi-collinearity test using VIF and TOL limits (Table 2) and analysis affirmed that all eighteen variables were suitable for evaluating the multi-hazard susceptibility in the study area.

**Table 2:** Multi-collinearity analysis for selected independent variables.

Variables	Collinearity test	
	VIF	Tol
Altitude	1.87	0.48
Slope	2.60	0.41
Aspect	0.90	0.93
Plan curvature	1.57	0.64
Profile curvature	1.44	0.69
Distance from river	1.46	0.68
Distance from road	1.38	0.72
Rainfall	1.47	0.68
Land use	1.54	0.65
Lithology	2.03	0.49
TWI	1.67	0.60
NDVI	1. 25	0.97
NDSI	1.39	0.67
MR	1.82	0.53
RR	2.06	0.50
SPI	2.21	0.43
STI	1.92	0.53

#### Index importance degree analysis

The importance of the geo-environmental variables for multi-hazard susceptibility mapping was estimated (Fig. 15). The most important variables for predicting FFS were rainfall (100%), altitude (81.89%), and distance from the river/streams (75.03%). For the LS the most important variables were slope (23.5%), lithology (18.7%), and elevation (18.3%). While the DF most important variables were Rainfall (25%), MR (21%), and RR (18%). Other variables were associated with moderate to lower importance.

**Fig. 6:**
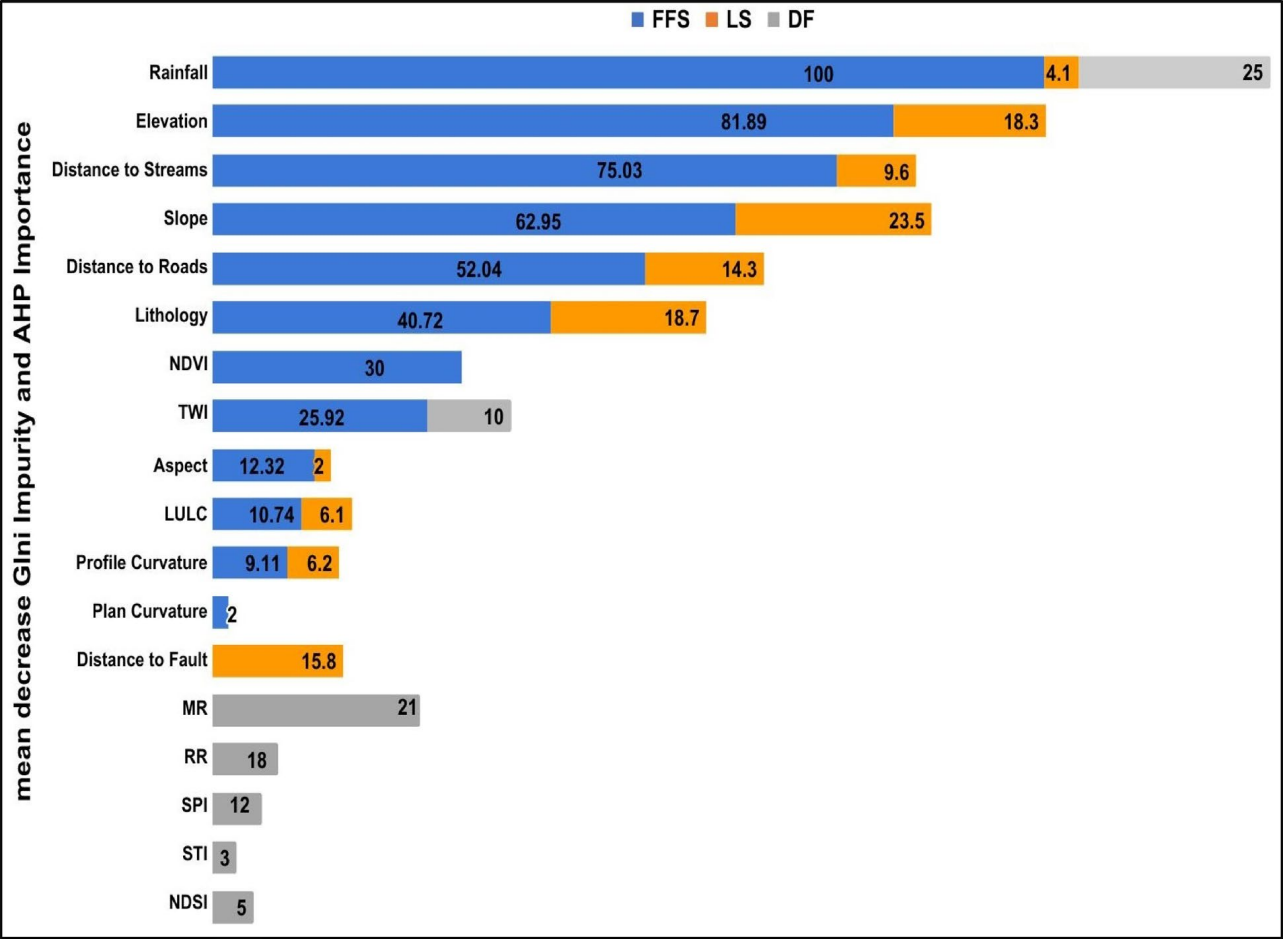
Relative importance of geo-environmental variables for individual hazard models, LS and FFS importance weights were derived from the mean decrease in Gini impurity of the RF, while DF variable importance was obtained from the weights assigned in the AHP model.

#### Landslide susceptibility

The areal coverage of LS zones covers an area of 20% (2335.84 km^2^) Very High LS, 25% (2826.42 km^2^) High LS, 14% (1614.55 km^2^) Moderate LS, 16% (1831.93 km^2^) low LS, and 24% (2776.02 km^2^) Very Low LS (Fig. 16). The very high and high susceptibility zones are located in places with steep slopes (> 30°) in the northern, northwestern, northeastern, and central parts. Proximity to fault lines (< 200 m) and rivers (< 300 m) also increases susceptibility. Further, factors contributing to elevated landslide risk include low vegetation cover, deforestation, and anthropogenic activities such as road construction and agricultural terracing. These factors, combined with the district’s rugged topography, make these areas highly prone to slope failures.

#### Flash flood susceptibility

The flood and non-flood points containing the values of flash flood conditioning factors were used as input datasets in Python (scikit-learn) to build FFS models. The areal coverage of very high to high FFS areas was 5% (536.306 km^2^) and 1% (131.43 km^2^), while the moderate, low, and very low susceptible zones and the areal coverage of these zones was 18% (1983.02 km^2^), 48% (5492.67 km^2^), and 28% (3157 km^2^), respectively (Fig. 16). The FFS map shows the regions associated with lower altitudes (1778–3553 m) and gentle slopes, specifically in the lower Hunza, central Hunza, and northwest and southwest regions along riverbanks are highly susceptible to frequent flash floods. This is due to intense rainfall events, glacier melt, and water accumulation. Land cover types like waterbodies, sparse vegetation, and built-up areas further increase flood risk in these regions. Conversely, regions with higher altitude (4311–7729 m) and steeper slopes reflect lesser susceptibility, as water moves away easily. However, these results show the combined impact of terrain features and hydrological factors on flood susceptibility in District Hunza.

#### Seismic hazard

The SH map classifies the district into: 11% (1288.63 km^2^) very high SH, 23% (2689.86 km^2^) high SH, 28% (3078.39 km^2^) moderate SH, 20% (2263.38km^2^) low SH, and 18% (2118 km^2^) very low SH (Fig. 16). The reclassification of terrain slope and Vs30 reflects that areas with higher Vs30 values, typically found in steeper slopes and harder rocks, have lower seismic hazards. In contrast, areas with lower Vs^30^ values, found in softer sediments and flatter terrain, are more prone to seismic hazards. These areas tend to amplify ground shaking during an earthquake. Notably, the central and northeastern regions, with softer sediments, show higher seismic hazards, making them more vulnerable. Regions near fault lines also face increased seismic risk due to lower Vs^30^ values, highlighting the impact of geology and topography on SH.

#### Debris flow hazard

The DF hazard map categorizes catchments into five hazard levels using the natural breaks (Jenks) method (Fig. 16). The lower part of the district contains catchments with very high to high hazard levels, characterized by steep terrain, high rainfall, and loose sediment. This combination increases susceptibility to debris flow initiation and propagation. The results indicate ten very high-hazard catchments, covering 2% of the district (252.26 km²), and nine high-hazard catchments, covering 5% of the district (598.48 km²). Additionally, ten moderate hazard catchments, spanning 9% of the district (1035.25 km²), are identified. Driven by terrain conditions and intense rainfall, these zones pose significant debris flow risks.

**Fig. 7:**
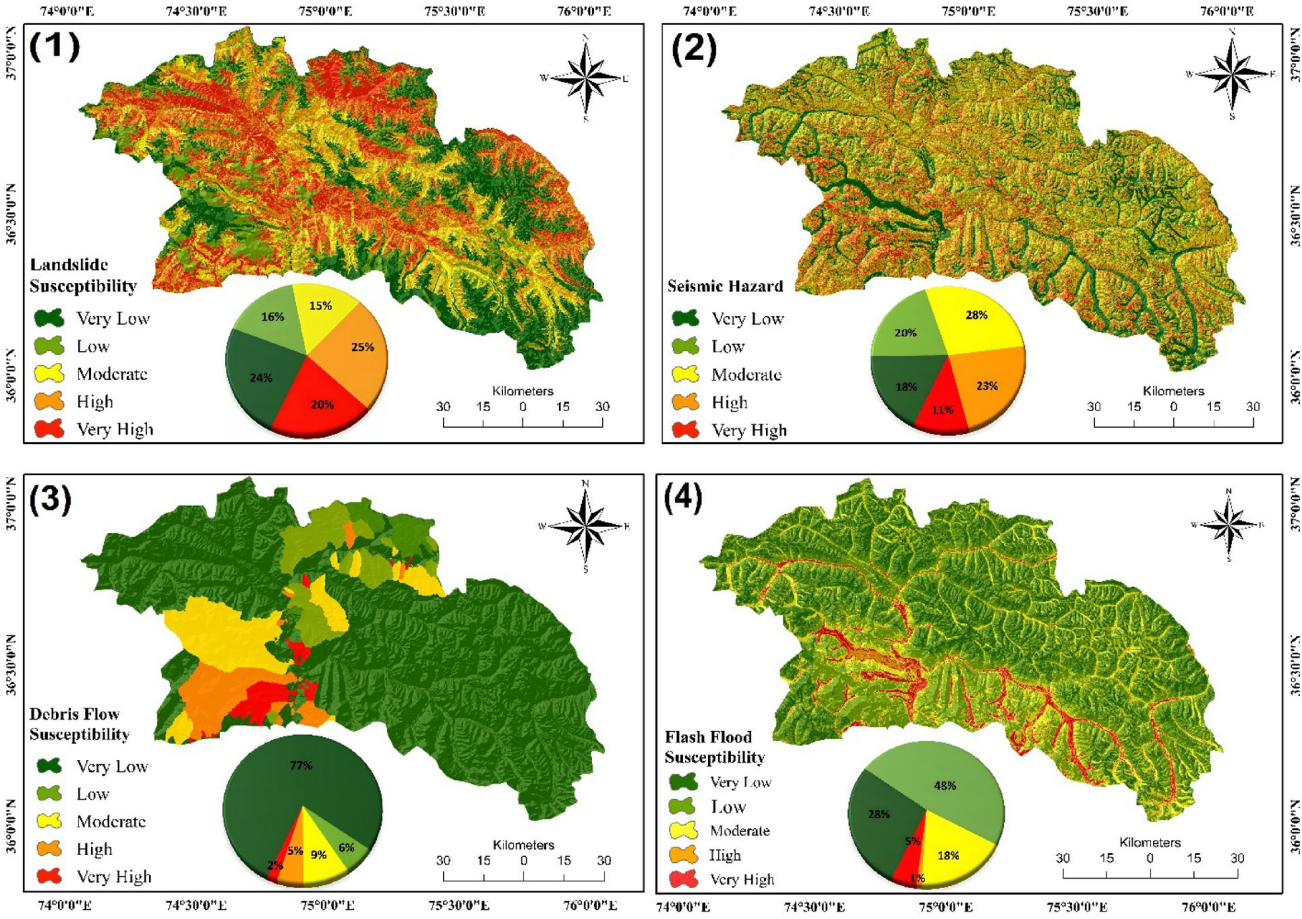
Susceptibility maps showing the four hazards: LS map generated through a ML-FBCR model, SH map created by reclassifying slope and Vs^30^ values, DF map developed using AHP, and FFS map generated through an ML- FBCR model.

### Multi-hazard analysis results

#### Index importance degree analysis

The MDG index, extensive field survey, available literature, local experts’ opinions, and engagement with decision-makers were considered to determine the importance of each hazard: SH was given (5.93) importance, LS (8.06), GLOF hazard (8.36), FFS (5.88), and DF (9.69).

#### A synthesized multi-hazard susceptibility map

All the selected hazards were integrated into a synthesized multi-hazard map. Pixels of the final integrated multi-hazard map were reclassified through ArcGIS Pro 2.8.3 natural break method into eight classes: Low Hazard, LS, FF, DF + FF, FF + LS+DF, SH + DF+LS + FF, DF + LS+GLOF + SH, and FF + SH+GLOF + LS+DF (Fig. 17), and the area were calculated for different classes of various hazards in percent and in km^2^ (Table 3).

**Fig. 8:**
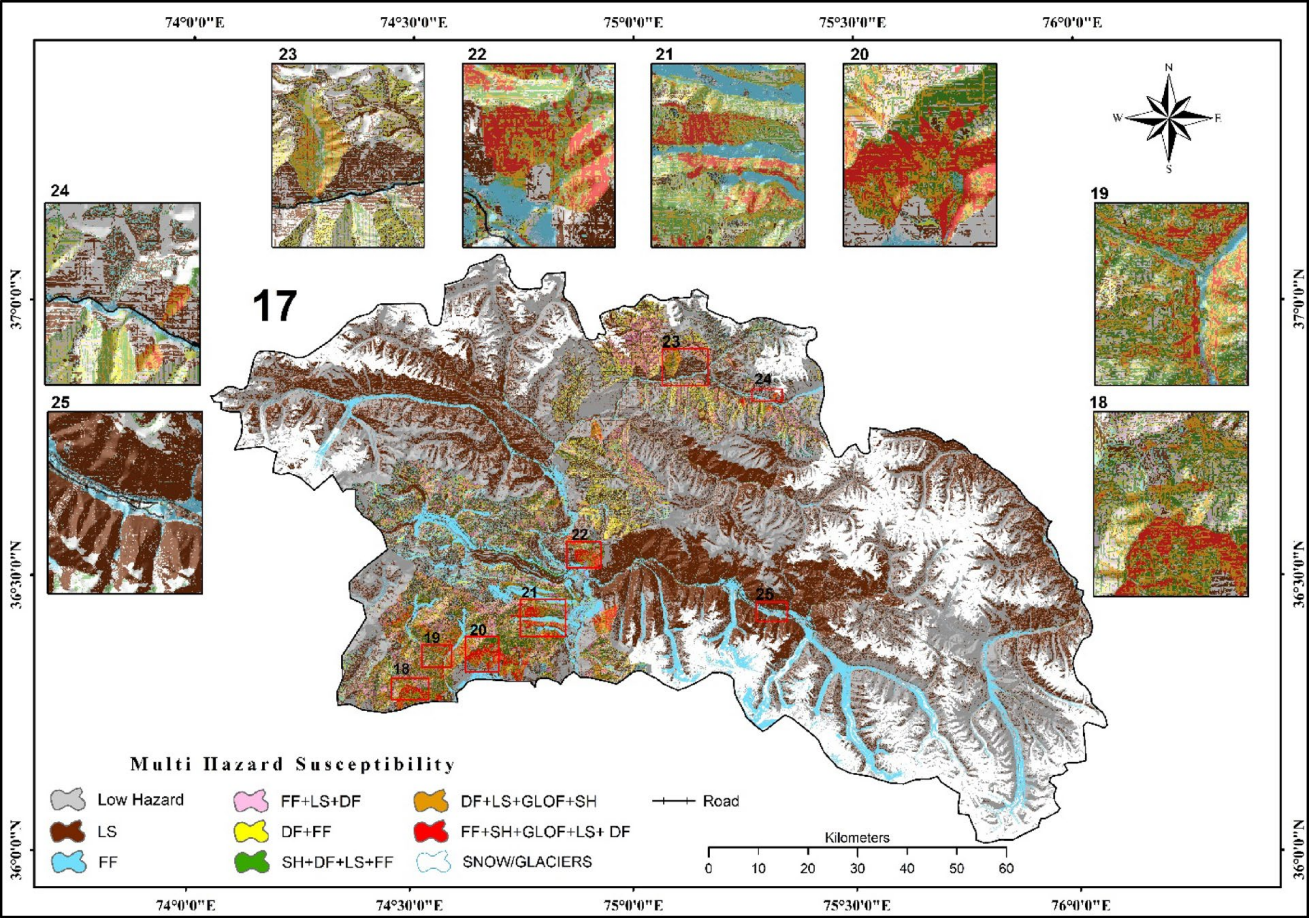
Multi-hazard susceptibility map produced using FBCR model, synthesizing the five hazard maps for district Hunza (LS Landslide, FF flash flood, DF debris flow, SH seismic hazard and GLOF), and visualized in ArcGIS Pro 2.8.3.

**Table 3:** Showing the areas of multi-hazard classes.

Multi-hazard	Area (km)^2^	%
Low hazard	6,469	56.84
LS	2630	23.11
FF	690.34	6.07
DF + FF	529.91	4.66
FF + LS+DF	453.11	3.98
SH + DF+LS + FF	327.66	2.88
DF + LS+GLOF + SH	190.07	1.67
FF + SH+GLOF + LS+ DF	91.29	0.80
Total	11380.95	100.00

Areas of only LS 2630 km^2^ (23.11%) or FF 690.34 km^2^ (6.07%), areas with DF + FF is 529.91 km^2^ (4.66%) and FF + LS+DF 453.11km^2^ (3.98%), while the areas of SH + DF+LS + FF 327.66 km^2^ (2.88%), DF + LS+GLOF + SH 190.07 km^2^(1.67%), FF + SH+GLOF + LS+DF 91.29 km^2^ (0.80%), and Low hazard 6469 km^2^ (56.84%). Significant variations can be seen in the integrated map in the northern part of Khunjerab Pass, especially in the NW and around Bara Khun Village and Pidakkesh Village (Fig. 17, Panels 23 and 24), where there is an overlap between multiple hazards such as debris flows, landslides, and flash floods. In the NW region and close to Bara Khun Village of Khunjerab Pass, seasonal livestock overgrazing has resulted in decreased vegetation cover, increased erosion, and degradation of the land. In addition, the lower part of District Hunza shows substantial variations and overlap in all five hazards including the villages of Murtazabad (Fig. 17, Panel 18), Hassanabad (Fig. 17, Panel 19), Karimabad and Aliabad (Fig. 17, Panel 20), and Hussaini (Fig. 17, Panel 21), while, the map shows that Shimshal Valley (Fig. 17, Panel 25) is mainly susceptible to landslides and flash floods. This is due to steep terrain, prevailing climatic conditions, intense rainfall, melting of glaciers, proximity to riverbanks, and human activities like infrastructure construction and population growth. The result indicates that no area is free from hazards even low-hazard areas are mainly found at higher altitudes and are primarily blanketed in snow/glaciers, showing that even these regions are susceptible to hazards.

#### Using out-of-bag estimates to monitor error

The results showed that the number of cycles increased from 250 to 500, and the number of trees in all MSE tests showed a decrease in every set of hazards from 29.44 to 28.09, respectively, as shown in Fig. 26. This accounts for a variation explaining percentage ranging from 3.65 to 1.86 for DF, 32.55 to 29.50 for FF, GLOF 9.64 to 7.52, LS 27.51 to 25.93, and the SH is 33.44 to 32.84. These percentages indicate how well the model captures the variability in each hazard, with higher values of 33.44 to 32.84 indicating a better fit of the model to the data.

**Fig. 9:**
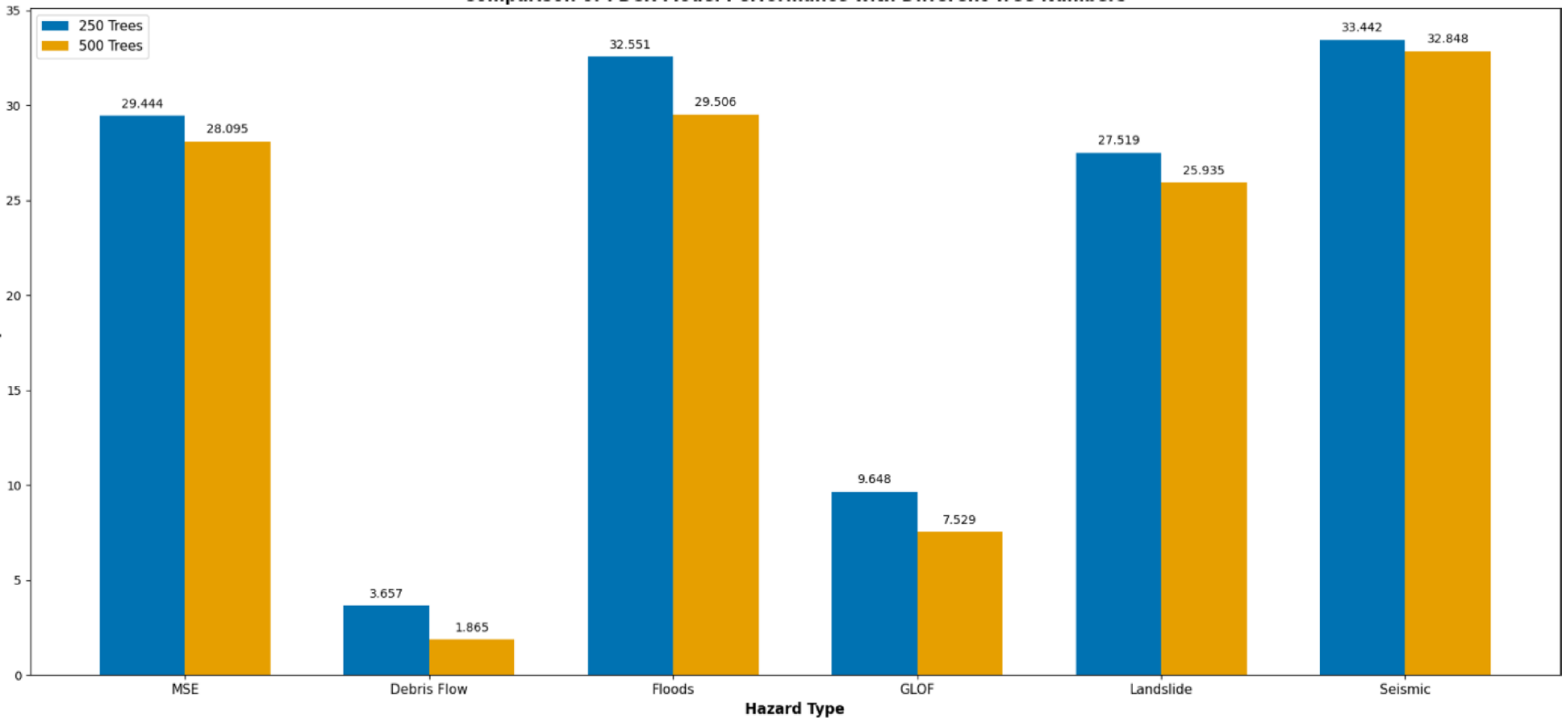
Showing the FBCR out-of-bag error with the number of decision trees.

## Model performance validation

The FBCR-model displayed the highest success-rate curve AUC value (0.866) for the synthesized multi-hazard map, the AUC value (0.822) for FFS map, while 0.843 value for LS and the AHP results with AUC value (0.791) for the DF.

## Discussion

Northern Pakistan is susceptible to hydro-meteorological and geological hazards making multi-hazard studies essential. The present study takes a crucial step forward by developing the synthesized multi-hazard map for district Hunza Northern Pakistan using an ML model^36^. This novel approach, integrated multiple-hazards-FFS, DF, LS, GLOF, and SH (Fig. 16) to advance disaster risk reduction efforts in Hunza district, northern Pakistan. Natural hazard research has traditionally concentrated on individual threats in isolation, ignoring the critical connections between them, this oversight may lead to miscalculations of risk^82^. By examining how multiple hazards intersect and overlap in a specific area, we can gain a deeper understanding of their collective risk than is yielded by simply combining the results of single-hazard^19^. For this purpose, procedures have been developed worldwide based on varying situations and with varying amounts of available data, but they have advanced the modeling process and have revealed the spatial distributions of the natural hazards in many study areas. In this study, FBCR-supervised ML model utilized to develop an integrated multi-hazard map in a mountainous region of northern Pakistan. Besides determining variable importance of each hazard, we employed an integrated approach including the mean decrease (Gini Impurity) of FBCR model, extensive field surveys, available literature, local experts’ opinions, and engagement with decision-makers. This approach yielded the following importance values for each hazard: Seismic hazard (5.938), Landslide Susceptibility (8.064), GLOF hazard (8.364), Flash Flood (5.888), and Debris Flow (9.694). Given the region’s geological and climatic characteristics, no area is completely hazard-free, with most regions susceptible to at least one hazard. A substantial portion of the district faces the threat of multiple hazards, with spatial overlap between flash flood, landslides, seismic, GLOFs, and debris flow hazards. Particularly, our study finds that, while some areas are susceptible to a single hazard, numerous other areas might face two or even all five-hazards simultaneously, underscoring the complexity and severity of the hazard in Hunza (Fig. 17, Panel 18). Furthermore, the results also highlight the evidence of spatial overlap of multiple hazards in low-lying locations, where the built-up area is primarily dispersed along lower elevations with scattered settlements^83^. The lower and central regions of the district appeared to be slightly more susceptible to multiple hazards due to the combination of increased constructed developments and climate events. Moreover, the Karakorum highway (KKH) and other essential road network is located in the Hunza district. The study confirms a multi-hazard susceptibility along the entire Karakorum highway. The FBCR model ROC-AUC value (0.866) indicates high performance and no overfitting, indicating it as an effective ML technique for multi-hazard susceptibility assessment^84^. These results are consistent with the study of other authors, Pourghasemi, et al.^14^ created an integrated map considering-landslides, foods, and earthquakes using the ensemble model called SWARA-ANFIS-GWO. They found 17.14% of the region is influenced by no hazard, and most parts were prone to landslide and flood hazards together (33.70%). They also asses’ accuracies of 84% and 80% for flood and landslide maps. Ahmad, et al.^18^ developed hazard inventories for upper Indus basin using remote sensing, field observations, and historical data to measure geohazards (such as landslides and debris flows) and then created a multi-hazard map using statistical methods, including SE, FR, WoE, and LR, the final susceptibility map shows the regions with overlapping hazards. Ullah, et al.^24^ developed a multi-hazard susceptibility map by combining flash flood, landslide, and debris flow in district Shangla using the deep learning algorithm coupled with-historical hazards data, topography, hydrology, and environmental data where the final multi-hazard map demarcates that 62.43% of region is exposed to hazards while 37.57% of the study area are hazard-free. Rehman, et al.^85^ have developed the database inventories for the geo-hazards (landslides and floods) and applied the AHP, frequency ratio and geospatial technique to produce a compound multi-hazard susceptibility map, and the map showed that majority of these geo-hazards were found in the high and very high susceptibility zones in Northwest Himalayas Pakistan. Further, integrated multi-hazard map will empower sustainable development and adaptive management in Hunza District by providing a comprehensive view of environmental hazards^86^. It will enable planners to implement integrated strategies, initiatives, and resource allocation, accounting all hazards simultaneously, for a resilient and sustainable future that it seems more susceptible to climate change, which is projected to intensify the frequency and harshness of extreme weather events (IPCC, 2014; Ministry of Climate Change, Pakistan, 2018). The synthesized multi-hazard map will also support prevention and mitigation programs that lower risk and improve resilience through policy and decision-making at the local, national, and international levels. Communities can identify intersections and cascade effects of natural disasters, develop comprehensive risk reduction strategies, and may help to raise awareness by identifying high-risk places. This strategy enhances emergency response planning, encourages involvement of stakeholders, and supports sustainable development. Additionally, we also believe that our findings will be useful for international readership because the developed maps and the approach will be used by relevant individuals or organizations involved in major construction projects in areas susceptible to multiple-hazards. However, this study also has several limitations such as lacks of glacier inventory data, reliant on past statistics, and lacks access to real-time data on hydro-meteorological hazards. The study’s spatial resolution might not be able to capture local-scale diversity, and the FBCR-model has a probability of overfitting^87^. Furthermore, multi-hazard susceptibility mapping was not as effective as it may have been since ML ignored the spatial structures of hazards while accounting for their spatial attributes. To address these problems, research on the integration of multi-hazard mapping using ML models combined with deep learning algorithms is being suggested^88^, which can make full use of both the spatial structure and attribute information of spatial objects. Secondly, integrated multi-hazard mapping has further limitations, with only one available GLOF map for the entire Hunza region, which restricts the accuracy of risk assessment. Therefore, this study recommends that further GLOF events should be generated to enhance the estimations of integrated hazard risks. In addition, the integrated multi-hazard map needs to be used to evaluate the exposure of elements that at risk, vulnerability of the region, and the total risk from the combination of integrated multi-hazard. Looking ahead, it is crucial to consider future exposure of elements at risk and develop strategies for a more resilient tomorrow.

## Conclusion

In this study, integrated multi-hazard modeling was conducted for Hunza District, Northern Pakistan, by generating a composite hazard inventory to construct an integrated multi-hazard susceptibility map. Five hazards were considered: FFS, DF, SH, (GLOF), and LS. By integrating these hazards, we developed an effective Integrated Multi-Hazard map which exposes the reality that a substantial portion of the district faces the threat of multiple hazards. Particularly, the central and lower parts of the district, areas along transportation corridors, demonstrate relatively higher multi-hazard susceptibility. These findings highlight the importance of adopting integrated hazard assessment approaches in mountainous environments where hazard interactions are common. Although this study focuses on susceptibility rather than direct risk quantification, the generated multi-hazard maps provide a useful spatial framework to support land-use planning, infrastructure development, and disaster risk reduction strategies. Incorporating multi-hazard perspectives into planning processes may contribute to more informed decision-making and improved resilience in regions exposed to complex hazard dynamics.

## Data Availability

All datasets generated for this research are provided in the manuscript, and external datasets from literature were included with proper permissions under the relevant licenses.
